# Synthesis of Phospholipid-Protein Conjugates as New Antigens for Autoimmune Antibodies

**DOI:** 10.3390/molecules200610253

**Published:** 2015-06-03

**Authors:** Arindam Maity, Claudia Macaubas, Elizabeth Mellins, Kira Astakhova

**Affiliations:** 1 Nucleic Acid Center, Department of Physics, Chemistry and Pharmacy, University of Southern Denmark, Campusvej 55, Odense 5230, Denmark; E-Mail: arindam.ju05@gmail.com; 2 Dr. B C Roy College of Pharmacy and AHS, Durgapur, West Bengal 713212, India; 3 Divisions of Human Gene Therapy and Pediatric Rheumatology, Program in Immunology, Stanford University School of Medicine, 269 Campus Drive, Stanford, MC 5164, USA; E-Mails: macaubas@stanford.edu (C.M.); mellins@stanford.edu (E.M.)

**Keywords:** CuAAC click chemistry, antiphospholipid syndrome, antigens, β2-glycoprotein I, phosphoethanolamine, prothrombin

## Abstract

Copper(I)-catalyzed azide-alkyne cycloaddition, or CuAAC click chemistry, is an efficient method for bioconjugation aiming at chemical and biological applications. Herein, we demonstrate how the CuAAC method can provide novel phospholipid-protein conjugates with a high potential for the diagnostics and therapy of autoimmune conditions. In doing this, we, for the first time, covalently bind via 1,2,3-triazole linker biologically complementary molecules, namely phosphoethanol amine with human β2-glycoprotein I and prothrombin. The resulting phospholipid-protein conjugates show high binding affinity and specificity for the autoimmune antibodies against autoimmune complexes. Thus, the development of this work might become a milestone in further diagnostics and therapy of autoimmune diseases that involve the production of autoantibodies against the aforementioned phospholipids and proteins, such as antiphospholipid syndrome and systemic lupus erythematosus.

## 1. Introduction

**Figure 1:**
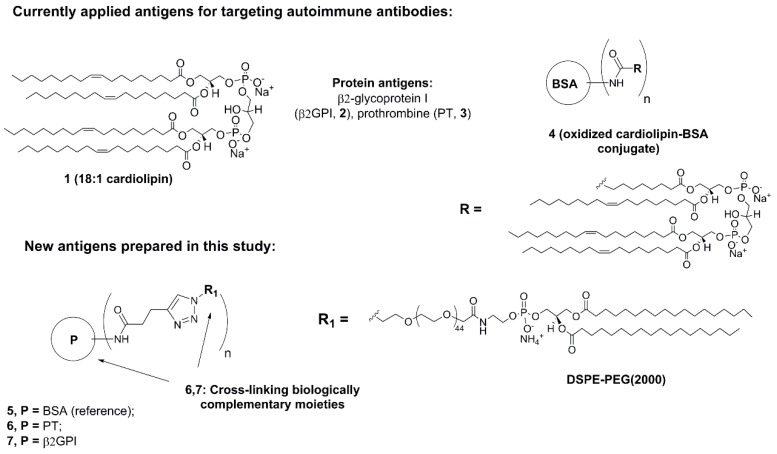
Antigens for detection of autoimmune Abs: CL, β2GPI, PT; general structure of the previously reported and new phospholipid-protein complexes **4**–**6**. BSA = bovine serum albumin, PT = prothrombin, β2GPI = β2-glycoprotein I, DSPE-PEG (2000) = 1,2-distearoyl-phosphoethanolamine polyethylene glycol-2000.

Autoimmune diseases are very diverse and can affect almost any part of the body, including the heart, brain, nerves, muscles, skin, eyes, joints, lungs, kidneys, glands, the digestive tract, and blood vessels [1,2]. Among such conditions, antiphospholipid syndrome (APS) is a multi-system autoimmune disorder characterized by arterial and/or venous thrombosis or recurrent fetal loss. APS is also characterized by presence of antiphospholipid antibodies (a-PL Abs, or a-PLs) [3,4,5,6]. In clinical practice, anti-cardiolipin antibodies (a-CL), anti-phosphoethanolamine (a-PE) and the lupus anticoagulant (LA) are the most established a-PL tests for the diagnosis of APS [7] (Figure 1). APS can also occur in isolation or in association with other autoimmune diseases such as systemic lupus erythematosus [8]. The clinical utility of routinely used a-PL assays have some uncertainty due to instability and heterogeneity of currently applied antigens [9]. Phospholipids are sensitive to oxidation and unstable upon freeze storage in solution. Moreover, new a-PLs may also expand the utility of the assay. This is especially important for diagnosis and monitoring of APS in patients with thrombosis and/or pregnancy morbidity, which are repeatedly negative for the currently used tests [10]. So non-criteria a-PL tests, like anti-prothrombin antibodies (a-PTs), are proposed to help in patients suspected of APS [11].

According to recent data, a-PLs bind not the phospholipids, but their complexes with human plasma proteins such as prothrombin (PT) and β2-glycoprotein I (β2GPI) [12,13,14,15,16]. This has motivated testing of phospholipid-protein complexes as antigens for diagnostics and studies of APS. In some novel assays the immunogenic phospholipid-protein complexes such as CL-β2GPI are generated non-covalently (by mixing the two molecules prior to the assay) [17]. However, non-covalent complexes are rather unstable which makes the assay hard to reproduce. Therefore covalent conjugation by NHS-chemistry has been proposed for bovine serum albumin (BSA) and other readily available “cargo” proteins with oxidized phospholipid molecules [18] (Figure 1, conjugate **4**).

For further development at this stage, in this paper we for the first time report on covalent binding of biologically complementary molecules, namely a synthetic PE derivative with human β2GPI and PT, via 1,2,3-triazole linker. Since its development by Meldal [19] and Fokin/Sharpless [20] the copper-catalyzed azide-alkyne cycloaddition (CuAAC) has become the important method for organic chemistry and synthetic biology. The ability to produce a stable linkage between alkyne and azide functional groups in different type of conjugation systems places CuAAC as a powerful tool in e.g., conjugation of oligonucleotides, peptides and proteins [21,22,23]. Herein, we demonstrate CuAAC chemistry as an improved method for preparation of novel phospholipid-protein conjugates with a high potential for the diagnostics of autoimmune diseases. The resulting phospholipid-protein conjugates show high binding affinity and specificity for the autoimmune Abs. The new molecules described herein are a promising tool for future studies and diagnostics of autoimmune conditions. Moreover, the developed CuAAC method can be applied for effective preparation of various phospholipid-protein complexes with potential applications in biochemistry and clinical studies of human diseases.

## 2. Results and Discussion

**Scheme 1:**
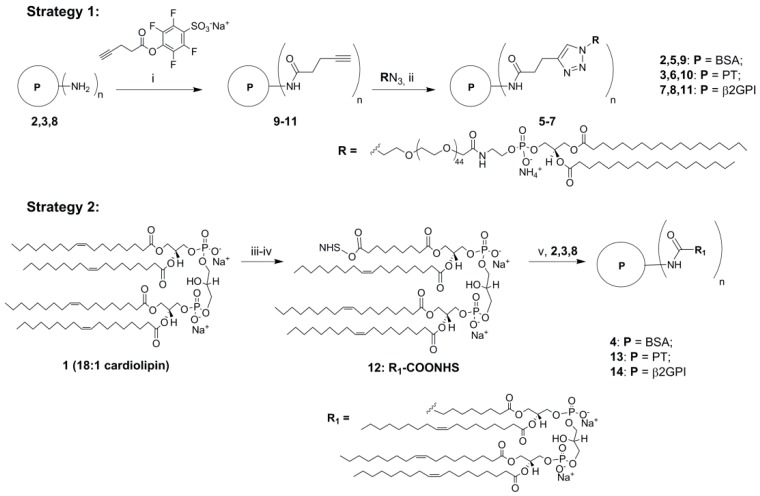
Synthesis of new phospholipid-protein conjugates by amide coupling and click chemistry methods. *Reagents and conditions*: (i) 0.1 M bicarbonate buffer–DMSO 9:1, +4 °C, 12 h; (ii) PE azide, CuSO4:TBTA 1:1.1, ascorbic acid, 1× PBS-DMSO–*t*-BuOH 3:2:0.1, *v*/*v*/*v*; (iii) KMnO_4_, NaIO_4_, *t*-BuOH–H_2_O 9:1, *v*/*v*, rt, 12 h; (iv) succinimide ester, *N*,*N*′-diisopropylcarbodiimide, DMSO, rt, 12 h; (v) proteins **2**,**3**,**8**, 0.1 M bicarbonate buffer–DMSO 9:1, *v*/*v*, rt, 12 h.

Previously phospholipids including oxidized CL have been attached to the protein cargos (BSA, lysocime *etc.*) by NHS-chemistry [18]. In our initial experiments we subjected natural CL **1** to oxidation in presence of KMnO_4_ and NaIO_4_ followed by DIC/NHS-mediated conjugation with BSA and for the first time PT and β2GPI (Scheme 1, Scheme S1, Supporting Information) [12,13,14,15,16]. According to chromatography, mass spectrometry and molecular modelling data, only one fatty acid residue of **1** was oxidized under the reaction conditions (Supporting Information, Scheme S1, Figure S1). However the applied procedure resulted in low yields, poor reproducibility and low diagnostic potential of the products (Supporting Information). Therefore, we developed a new CuAAC click approach for the attachment of synthetic PE antigen to clinically relevant β2GPI and PT (Scheme 1). In spite of structural differences, both CL and PE are immunogenic and proved to be useful in diagnostics of a-PLs [14]. The developed click procedure has advantages of high yields and purity of the products with improved chemical stability compared to oxidized phospholipids. Moreover, the new procedure can be directly employed for similar quantities of different phospholipids and proteins. In order to compare with previously reported analogues, we prepared a PE-BSA conjugate as well (Scheme 1).

First, we functionalized each protein with water soluble alkyne activated ester alkyne group [24] and then subjected it to CuAAC click reaction with commercially available PE azide in a molar ratio 1:25. After simple precipitation, we obtained the desired conjugated **5**–**7** in good yield and purity (Scheme 1; yields ≥ 80%; full conversion as determined by gel electrophoresis, Supporting Information, Figure S2).

The number of phospholipid groups per protein was estimated based on the difference in mass of the conjugate *vs.* unmodified protein using MALDI TOF mass spectrometry (Supporting Information, Figure S3). We observed that the highest number of PE residues was attached to BSA (*n* = 8–9), whereas 5–8 residues were linked to β2GPI and PT. We speculate that the 583 amino acid long globular BSA might have highest amount of amino groups on its surface available for functionalization. However, human β2GPI and PT (233 and 655 amino acids, respectively) have only few positively charged stretches on the surface [12]. According to the literature, these 5–7 amino acid long Lys/Asn-rich sequences are responsible for binding phospholipids by the two immunogenic proteins in biofluids [12]. Using oxidized CL we achieved conjugation of only 2–4 phospholipid residues to same proteins (Supporting Information). This could result from steric hindrance and low efficacy of DIC/NHS-mediated conjugation of corresponding biomolecules compared to click chemistry.

**Figure 2:**
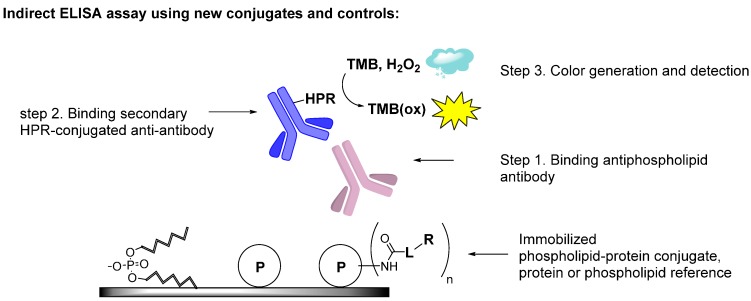
Representative scheme of enzyme-linked immunosorbent assay (ELISA) for detection of Abs against antigens used in this study: phospholipids, proteins and phospholipid-protein conjugates. P = protein, L = linker, R = phospholipid residue, TMB = 3,3ʹ,5,5ʹ-tetramethyl benzidine , HPR = horseradish peroxidase.

We applied conjugates **5**–**7** and control antigens in enzyme-linked immunosorbent assay (ELISA), using series of disease-associated or human normal plasmas (HNP, *n* = 10; Figure 2, Table 1). The disease-associated samples contained high levels of a-PLs, a-β2GPIs; control samples to assess cross-reactivity contained Abs to single-stranded and double-stranded DNA. These Abs cross-bind PL antigens, which negatively affects the assay specificity (Table 1).

Initially, Bradford analysis confirmed similar total protein levels in the plasma samples (40–50 mg/L), whereas rheumatoid factor (RF) IgG+IgA+IgM ELISA test showed negative results [25]. Thus, the influence of different protein levels and RF on the assay results was considered to be minimal. Furthermore, we slightly adjusted conditions of ELISA experiments for proteins, phospholipids and their conjugates (Experimental section, a-ssDNA and a-dsDNA) [14,15,16,17] Having done this, we examined a linear range of the assay for IgG and IgM Abs within plasma dilution range 1:80–1:500 [26]. Dilution 1:100 was found to be optimal for testing, whereas linear range was optimal for synthetic conjugates (Supporting Information, Figure S4). In agreement with previous reports, we found IgG Abs to be the most relevant for a-PL detection [27,28,29] (Table 1; Supporting Information, Table S1). Among other antigens, PE-PT and PE-β2GPI bound target Abs with highest signal to noise ratio compared to healthy controls. In turn, CL, PE-BSA and conjugates containing oxidized CL showed high number of false positive signals, most likely due to instability and lack of specificity discussed below (Supporting Information, Table S1, Figure S5). Therefore, in spite of the highest a-PL binding signal among tested molecules (absorbance 1.97), CL itself is of a rather poor value for diagnostic purposes and studies on autoimmune diseases [30,31,32,33].

**Table 1:** Results of IgG ELISA assay using controls and conjugates prepared in this study *.

Antigen	Absorbance at 450 nm: Analyte				
	a-PL	a-β2GPI	a-ssDNA	a-dsDNA	HNP (*n* = 10)
CL	1.97	0.90	0.90	0.63	0.45
β2GPI	0.75	1.01	0.60	0.32	0.22
CL:β2GPI ^§^	0.98	0.80	0.55	0.61	0.52
PT	0.45	0.31	0.43	0.45	0.23
BSA	0.31	0.25	0.34	0.28	0.24
PE azide	1.03	0.91	0.80	0.54	0.33
**5** (BSA-PE)	1.44	0.56	1.44	1.21	0.43
**6** (PT-PE)	1.05	0.44	1.01	0.72	0.37
**7** (β2GPI-PE)	1.02	1.15	0.45	0.34	0.19

* a-PL, a-ssDNA and a-dsDNA = human plasma tested highly positive against phospholipids, single-stranded and double-stranded DNA, respectively; a-β2GPI = monoclonal Ab against β2GPI. HNP = human normal plasma; averaged absorbance for n patients is presented (Δ ± 0.20). ^§^ β2-Glycoprotein I (β2GPI; 0.001%) was added to CL under blocking conditions resulting in non-covalent binding. CL = CL, PT = prothrombin. Each sample was measured in the duplicate with resulting deviation in absorbance Δ ± 0.20.

High cross-reactivity of a-PLs with other antigens such as ssDNA and dsDNA is another important obstacle for their utility in studies and diagnostics of autoimmune diseases [30,31,32,33]. As can be seen from data presented in Table 1, CL shows high binding affinity to other mono- and polyclonal controls including a-β2GPI, a-ssDNA and a-dsDNA. In this work we aimed at improved specificity of a-PL binding by covalent cross-linking of biologically complementary molecules such as PE with PT and PE with β2GPI. Our IgG ELISA experiments show that this has been achieved for PE-β2GPI conjugate (absorbance 1.02 *vs.* 0.45 and 0.34 when incubated with a-PL, a-ssDNA and a-dsDNA, respectively, compared to CL: 1.97, 0.90 and 0.63). Notably, attachment of PE to BSA and oxidized CL derivatives showed complete lack of discrimination for binding a-PLs and aDNAs (Table 1; Supporting Information). This additionally confirms our hypothesis that cross-connection of biologically complementary β2GPI and PE improves binding specificity of the conjugate.

**Figure 3:**
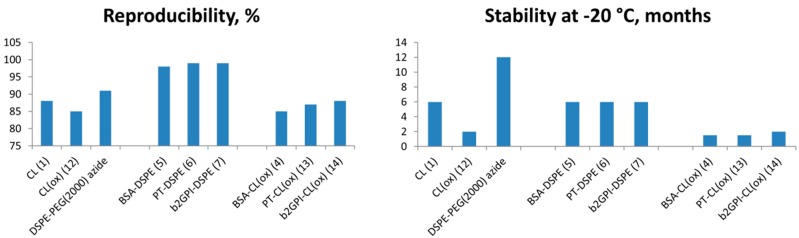
Comparative performance of phospholipid antigens in ELISA detection of autoimmune Abs. The reproducibility assay was performed independently three times over 6 week time period using controls: a-PL, a-β2GPI, a-ssDNA, a-dsDNA and HNP (*n* = 10).

As a final aspect, we evaluated reproducibility of ELISA tests and stability of antigens upon storage in solution at −20 °C (Figure 3) [34]. The latter was done by TLC and gel electrophoresis (Experimental section). As one can see, conjugates **5**–**7** showed superior reproducibility then individual phospholipids and oxidized CL conjugates (97%–98% *vs.* 83%–89%, respectively). Stability upon storage in solution was increased up to 6 months at −20 °C *vs.* 1.5–2 months for oxidized CL analogues. This implies that high purity and immunogenicity of the novel molecules has a positive effect on their diagnostic performance, which makes them promising tools for further studies of diverse autoimmune conditions [35,36]. In particular, absence of double bonds in the phospholipid’s fatty acid chain might have a positive effect on stability upon freezing the antigens [37].

## 3. Experimental Section

18:1 cardiolipin (1ʹ,3ʹ-bis[1,2-dioleoyl-*sn*-glycero-3-phospho]-*sn*-glycerol (sodium salt)) and PE azide (distearoyl-sn-glycero-3-phosphoethanolamine-*N*-[azido(polyethulene glycol)-2000] (ammonium salt), or DSPE-PEG(2000)) were purchased from Avanti Polar Lipids and used as received. β2-Glycoprotein I (β2GPI), bovine serum albumin (BSA) and prothrombin (PT) were obtained from Diarect Antigens and Sigma. Pentynoic sulfotetrafluorophenyl (STP) ester, reagents and solvents for click chemistry were provided by Lumiprobe. Other reagents, buffers and solvents (KMnO_4_, NaIO_4_, TMB, HPR anti-human IgG/IgM conjugates, acetate buffer (pH 5.6), PBS (pH 7.4), DMSO, Tween-20, H_2_SO_4_) were obtained from Sigma Aldrich (St. Louis, MO, USA) and used without additional purification.

96-well Maxisorb NUNC microplates were purchased from Thermofisher Scientific (Santa Clara, CA, USA). **Plasma controls** were obtained from Immunovision and Stanford University Hospital, Stanford, CA, USA. Monoclonal Ab against β2GPI was purchased from Diarect antigens. All the samples were tested rheumatoid factor (RF) negative (IgG+IgM+IgA), using corresponding diarect RF ELISA kit. **Bradford assay** was performed following manufacturers protocol using standard reagents (BioRad, Richmond, VA, USA) [38].

**Click reactions** were performed in 1.5 mL eppendorf tubes under argon. Starting protein was dissolved in fresh 0.1 M bicarbonate buffer (0.2 mg in 180 μL, pH 8.5) in 1.5 mL plastic Eppendorf tube. Fresh solution of pentynoic STP ester (15 μL of 1 mg/mL solution in DMSO:0.1 M bicarbonate buffer 1:1, *v*/*v*) was added and the reaction was kept at +4 °C overnight. The alkyne-labelled protein was precipitated from cold acetone, washed twice with acetone and dissolved in 1× PBS (300 μL). DMSO (160 μL), corresponding PE azide (5 μL, 1 mM solution in *t*-BuOH), ascorbic acid (10 μL of 25 mM freshly prepared stock solution) and Cu(II)-TBTA equimolar complex (25 μL of 10 mM stock solution) were subsequently added. The resulting mixture was deaerated, tightly closed, gently mixed and left at rt overnight. The product was afterwards precipitated from cold acetone and subsequently washed with acetone two times. The resulting conjugates were analyzed by mass spectrometry and gel electrophoresis. Final yields of products based on the absorbance at 280 nm: 88% (**5**), 82% (**6**), 80% (**7**).

**ELISA assay**. Coating of 96 well ELISA plates was performed overnight using 2 µg/mL solution of a corresponding protein or conjugate in 1× PBS (100 µL per well). In the case of coating with phospholipid-protein conjugates the plates were kept in dark at +4 °C overnight [39]. After washing 2 times with 1× PBS (300 µL), the plates were blocked for 1 h at RT with PTB buffer (50 µL Tween-20, 4 g BSA per 200 mL 1× PBS; 100 µL per well). Plates were washed 2 times with 1× PBS (300 µL) and incubated with plasma for 1.5 h at rt (100 µL per well). Plasma were diluted 1:100 using freshly prepared diluent (1 g BSA, 200 µL Tween-20 in 1 L 1× PBS). After washing 3 times with 1× PBS (300 µL) the second incubation was performed with a corresponding HPR-Ab (1.5 h, rt, 100 µL per well). The HPR conjugate (a-IgG or a-IgM) was diluted 1:20.000 in the same diluent as for the initial incubation. Plates were washed 3 times with 1× PBS (300 µL) and incubated with fresh TMB solution (100 µL/well) in 0.1 M acetate buffer (pH 5.4) for 15 min (100 µL/well). In doing this, 3 mg TMB was dissolved in 5 mL DMSO and then diluted to 50 mL with 0.1 M acetate buffer containing 3 µL conc. H_2_O_2_. The reaction was stopped with 1 M H_2_SO_4_ (50 µL/well). Plates were analyzed using Magellan TECAN microplate reader by measuring absorbance at 450 nm.

Coating of 96-well ELISA plated with cardiolipin was performed overnight at +4 °C (50 µg/mL in ethanol; 50 µL/well). The subsequent steps were similar to the protocol described above, except for using PTB containing a reduced amount of Tween-20 (10 µL/200 mL PTB), and, for selected plates, adding β2GPI to PTB (50 µL of 0.2 mg/mL solution per 10 mL PTB) [14,15,16,17].

**Reproducibility** of assays was evaluated by running three independent experiments using a series of pre-coated plates [14,15,16,17]. Each assay was performed independently over 6 week time period using controls a-PL, a-β2GPI, a-ssDNA, a-dsDNA and HNP (*n* = 10).

**Stability of antigens** upon storage at −20 °C was evaluated by analytical thin layer chromatography for phospholipids (Kieselgel 60 F254 precoated aluminium plates (Merck, Copenhagen, Denmark); solvent: chloroform:methanol:water 60:35:5, *v*/*v*/*v*), or gel electrophoresis for phospholipid-protein conjugates (SDS-PAGE using Coomassie stain).

## 4. Conclusions

In summary, we have developed a new procedure for the effective conjugation of proteins with phospholipids by CuAAC click chemistry, which gives high yields of the desired conjugates with advantages of high stability and easy purification. This procedure will allow a new systematic approach to the generation of phospholipid-protein complexes mimicking biologically active natural analogues. As demonstrated in this paper, the product complexes might become new useful tools for diagnostics and studies of human autoimmune diseases.

## Supplementary Materials

Supplementary materials can be accessed at: http://www.mdpi.com/1420-3049/20/06/10253/s1.
