# Supplementary material for: Synthesis of Phospholipid-Protein Conjugates as New Antigens for Autoimmune Antibodies
Source: Molecules. 2015 Jun 3;20(6):10253–63. doi: 10.3390/molecules200610253 (PMC6272759; doi:10.3390/molecules200610253)
Supplement: Supplementary file 1 [file molecules-20-10253-s001.pdf]

Supporting Information

Synthesis of CL-COONHS (12)

To a solution of cardiolipin (CL) **1** (10 mg, 6.66 μmol) in 1.5 mL tBuOH 100 μL aq. solution of $NaHCO_3$ (10 mg in 100 μL) was added. Then the solutions of $NaIO_4$ (30 mg in 200 μL water) and $KMnO_4$ (10 mg in 200 μL water) were subsequently added. The reaction was mixed for 3 h and kept for 24 h at rt in dark. The reaction was controlled by TLC. After starting material was detected by TLC the reaction was quenched by adding 150 mg Na_2SO_3. After stirring for 5 min the reaction become colorless and two liquid phases separated. The mixture was acidified with 5% HCl til pH 3.0 and washed twice with *t*-BuOH. *t*-BuOH fraction was dried over Na_2SO_4, evaporated *in vacuo*. Yield 70%; R_f 0.37 (chloroform:methanol:water 3:1.5:0.2, *v*/*v*/*v*), HRMS-ESI *m*/*z*: 1406.84772 ($[M + Na]^+$, $C_{72}H_{130}Na_2O_{19}P_2$ calcd 1406.84766).

To a solution of oxidized CL (5 mg) in 1 mL DMSO 4 mg succinimide ester in 20 μL DMSO and 8 μL *N*,*N*′-diisopropylcarbodiimide were added. After keeping the reaction at rt overnight TLC showed complete conversion of the starting material. The product was analyzed by HRMS-ESI and used in further steps without purification. R_f 0.64 (chloroform:methanol:water 3:1.5:0.2, *v*/*v*/*v*), HRMS-ESI *m*/*z*: 1503.86409 ($[M + Na]^+$, $C_{76}H_{133}NNa_2O_{21}P_2$ calcd 1503.86405).

According to TLC, a mono-oxidized CL **12** was exclusively formed. Having analyzed previous reports on CL structure and having built a molecular model, we concluded that the most sterically accessible group has been oxidized (Scheme S1, Figure S1).

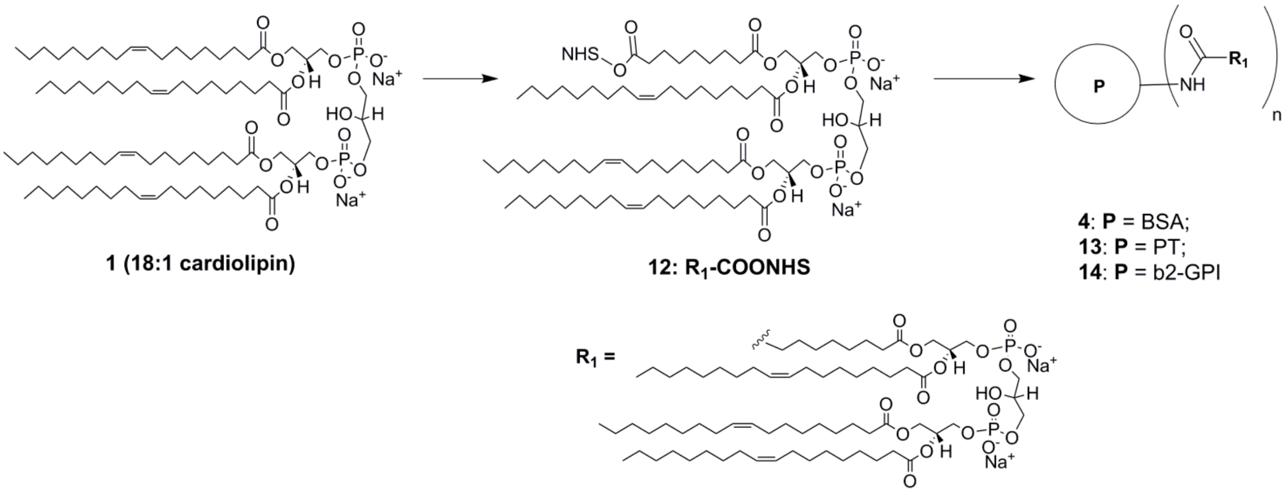

Scheme S1. Synthesis of oxidized cardiolipin (CL) and its protein conjugates.

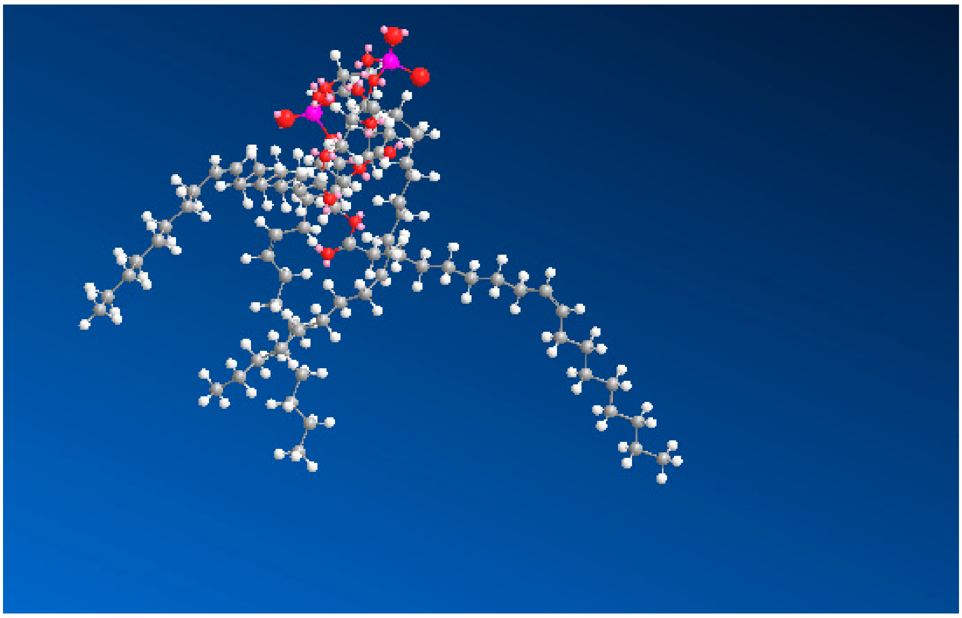

Figure S1. Molecular model of cardiolipin **1**. The model was build using MacroModel V9.1., and minimized using the Polak-Ribiere conjugate gradient method, the all-atom AMBER force field, and GB/SA solvation model [1].

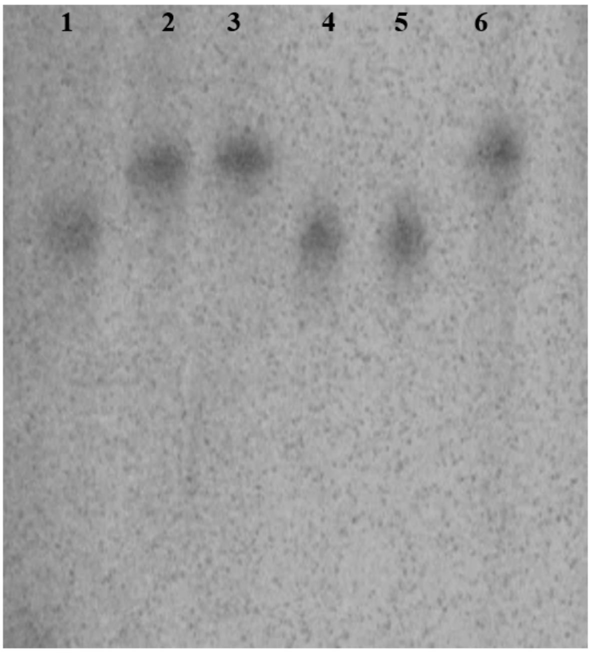

Figure S2. Representative SDS-PAGE gel electrophoresis of starting proteins (PT, BSA, lanes 1 and 4, respectively) and product conjugates **13** (lane 2), **6** (lane 3), **4** (lane 5), **5** (lane 6), using Coomassie stain.

Synthesis of Phosoholipid-Protein Conjugates (4, 13, 14)

Starting protein was dissolved in fresh 0.1 M bicarbonate buffer (0.2 mg in 180 µL, pH 8.5) in 1.5 mL plastic Eppendorf tube. Cardiolipin NHS-ester (20 µL of solution obtained in previous step) was added and the reaction was kept at rt overnight. The cardiolipin-labelled protein was precipitated from cold acetone, washed twice with acetone and re-dissolved in 200 µL of 1× PBS.

MALDI MS results (conjugate/unmodified protein, kDa (estimated amount of attached modifications, *n*)): **4**/BSA, 71.0–72.8/66.4 (*n* = 3–4); **13**/PT, 76.6–84.4/72.0 (*n* = 3–5); **14**/β2GPI, 53.1–55.0/50.0 (*n* = 2–3);

5/BSA, 88.7–90.3/66.4 (*n* = 8–9); **6**/PT, 88.0–91.6/72.0 (*n* = 6–7); **7**/β2GPI, 63.5–67.3/50.0 (*n* = 5–6).

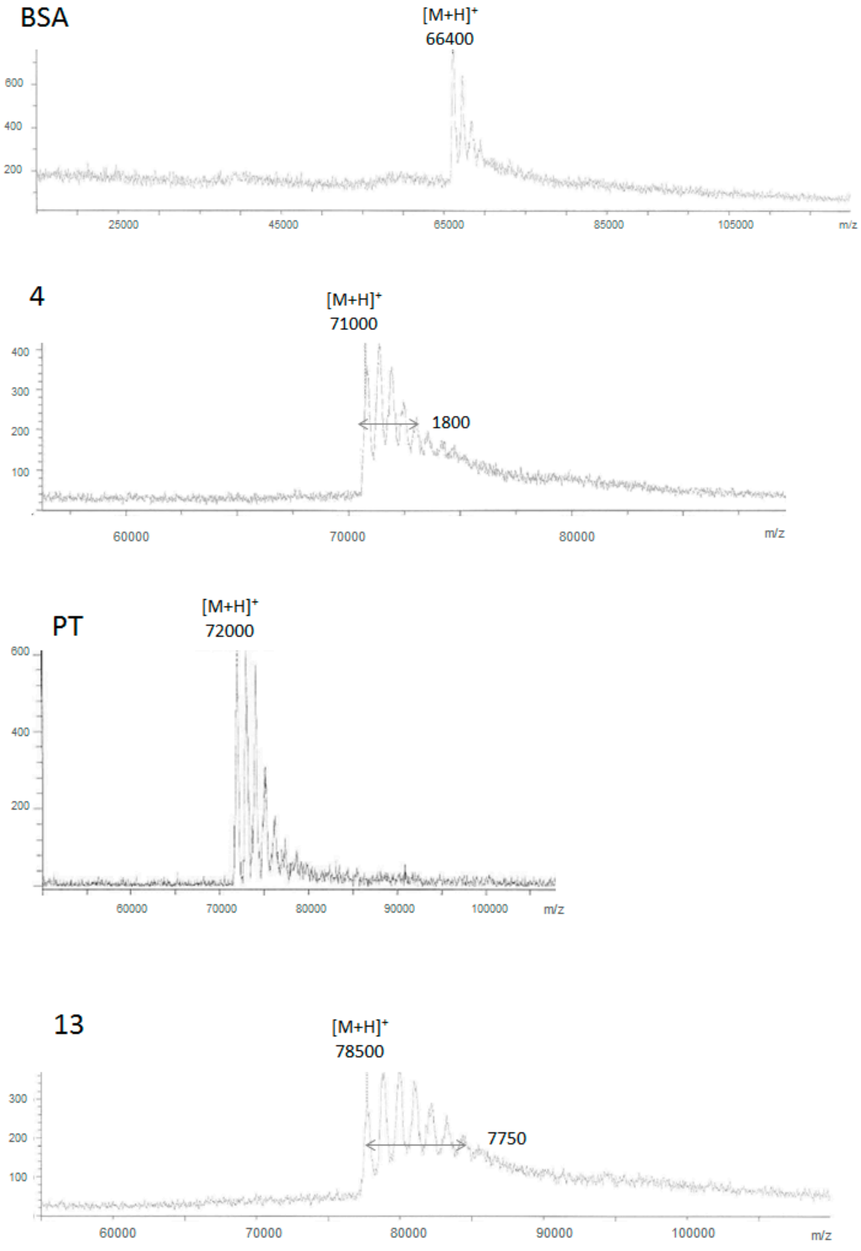

Figure S3. *Cont.*

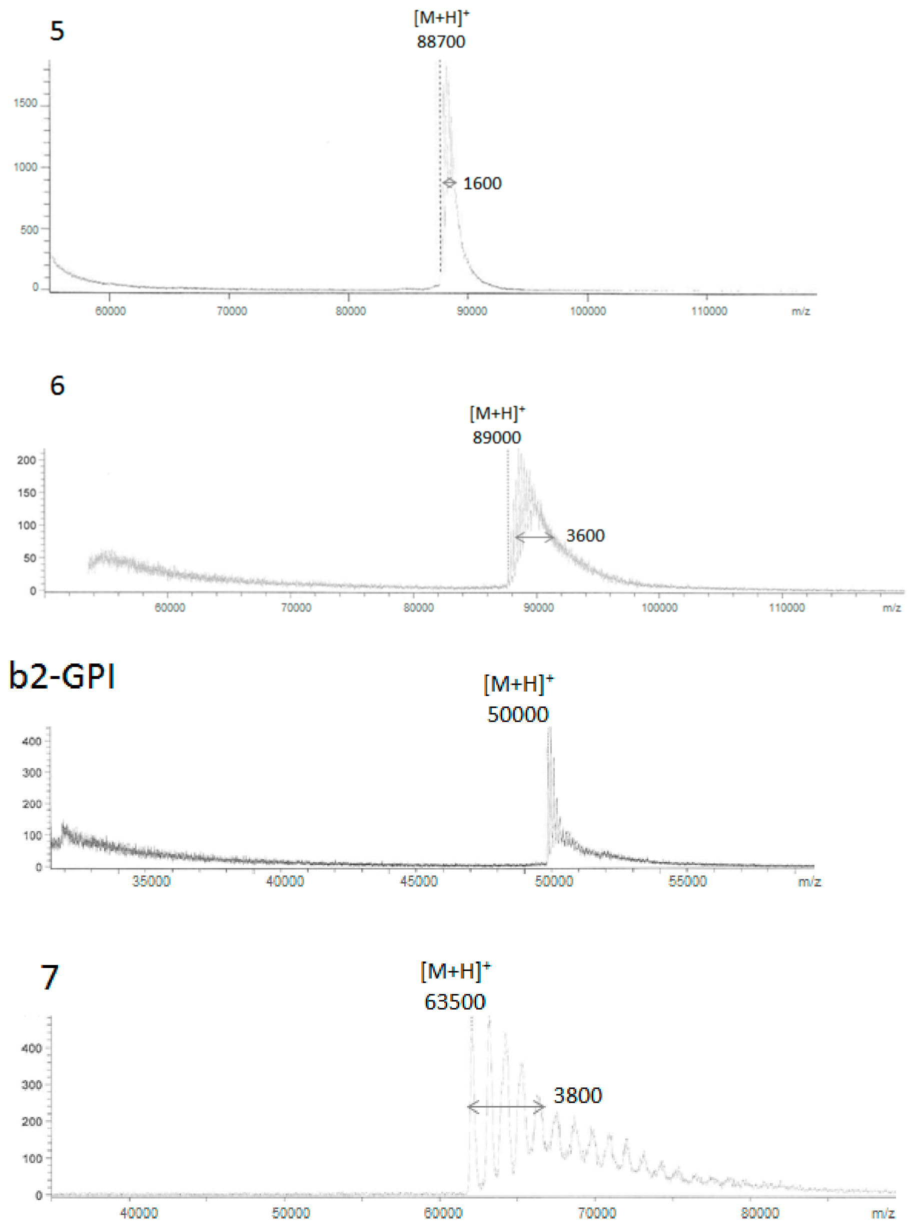

Figure S3. Representative mass spectra of proteins and phospholipid-protein conjugates prepared in this study. Double-headed arrows indicate approximate peak widths at half-height for the corresponding conjugates.

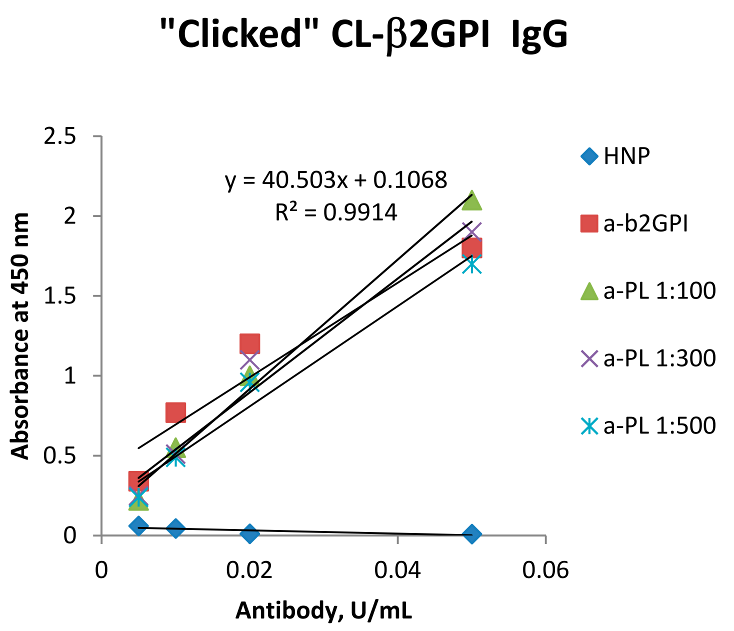

(A)

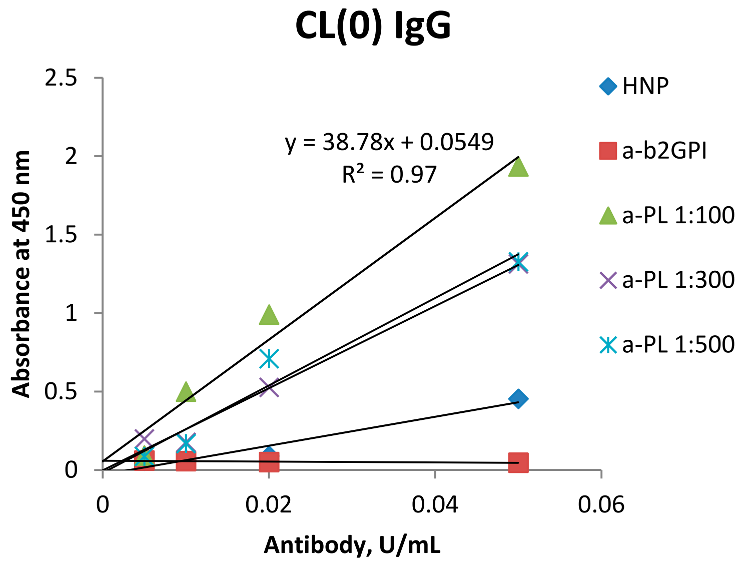

(B)

Figure S4. Representative determination of linear range of IgG ELISA assay using conjugate **7** (**A**) and unmodified CL (**B**). HNP = human normal plasma; a-β2GPI and a-PL are controls containing antibodies toward human β2-GPI and PLs.

Table S1. Results of IgM ELISA assay using controls and conjugates prepared in this study *.

Antigen	Absorbance at 450 nm IgM				
	**a-PL**	**a-β2GPI**	**a-ssDNA**	**a-dsDNA**	**HNP (*n* = 10)**
CL	0.65	0.11	0.45	0.32	0.11
β2GPI	0.52	0.55	0.43	0.21	0.32
CL:β2GPI §	0.50	0.45	0.39	0.15	0.40
PT	0.33	0.20	0.50	0.14	0.22
BSA	0.22	0.28	0.43	0.33	0.20
PE azide	0.40	0.10	0.32	0.11	0.09
**5 (BSA-PE)**	0.82	0.33	0.68	0.22	0.65
**6 (PT-PE)**	1.01	0.24	0.79	0.15	0.74
**7 (β2GPI-PE)**	0.99	1.11	0.82	0.22	0.26

§ β2GPI (0.001%) was added to CL under blocking conditions resulting in non-covalent binding. * a-PL, a-ssDNA and a-dsDNA = human plasma tested highly positive against phospholipids; single-stranded and double-stranded DNA, respectively; a-β2GPI is a monoclonal antibody towards β2-glycoprotein I (β2GPI). HNP = human normal plasma; averaged absorbance for n patients is presented (Δ ± 0.20). CL = cardiolipin, PT = prothrombin. Each sample was measured in the duplicate with resulting deviation in absorbance Δ ± 0.20.

Table S2. Results of IgG and IgM ELISA assay using conjugates **4**, **13** and **14** containing oxidized cardiolipin *.

Antigen	Absorbance at 450 nm IgG (IgM)				
	**a-PL**	**a-β2-GPI**	**a-ssDNA**	**a-dsDNA**	**HNP (n = 10)**
**4 (BSA-CL)**	0.83 (1.12)	0.36 (0.43)	0.82 (0.70)	0.21 (0.52)	0.51 (0.89)
**13 (PT-CL)**	0.71 (0.89)	0.44 (0.44)	1.01 (0.69)	0.72 (0.45)	0.45 (0.65)
**14 (β2GPI-CL)**	0.77 (0.88)	1.21 (0.67)	0.54 (0.80)	0.47 (0.32)	0.55 (0.54)

* For details see Tables 1 and S1.

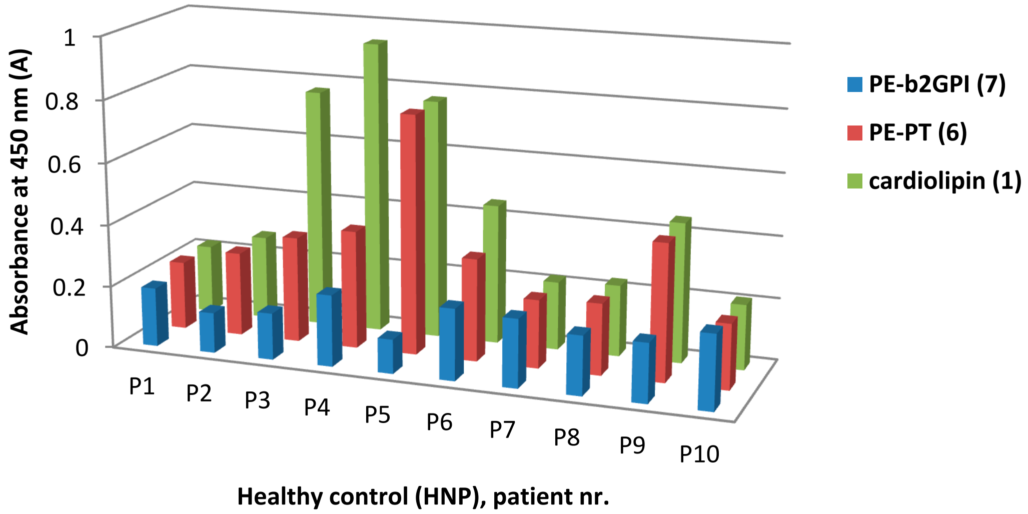

Figure S5. Results of IgG ELISA assay of healthy control samples (HNP, n = 10) using CL and conjugates **6**–**7**. Weakly positive result: absorbance (A) > 0.4; medium range positive result: A > 0.65.

Reference

1. Jørgensen, A.S.; Gupta, P.; Wengel, J.; Astakhova, I.K. "Clickable" LNA/DNA probes for fluorescence sensing of nucleic acids and autoimmune antibodies. *Chem. Commun.* **2013**, *49*, 10751–10753.
